# Europe’s renewable energy directive poised to harm global forests

**DOI:** 10.1038/s41467-018-06175-4

**Published:** 2018-09-12

**Authors:** Timothy D. Searchinger, Tim Beringer, Bjart Holtsmark, Daniel M. Kammen, Eric F. Lambin, Wolfgang Lucht, Peter Raven, Jean-Pascal van Ypersele

**Affiliations:** 1 Woodrow Wilson School of Public and International Affairs, Princeton University, Princeton, 08544 New Jersey USA; 2 Integrative Research Institute on Transformations of Human Environment Systems (IRI THESys), Humboldt-Universität zu Berlin, Berlin, 10099 Germany; 3 Statistics Norway, Oslo, N-0131 Norway; 4 Energy and Resources Group, Renewable and Appropriate Energy Laboratory, and Goldman School of Public Policy, UC Berkeley, Berkeley, 94720 California USA; 5 School of Earth, Energy & Environmental Sciences and Woods Institute for the Environment, Stanford University, Stanford, 94305 California USA; 6 Earth and Life Institute, Université catholique de Louvain, B-1348, Louvain-la-Neuve, Belgium; 7 Potsdam Institute for Climate Impact Research, Potsdam, 14473, Germany; 8 Humboldt-Universität zu Berlin, 10099, 8 Berlin, Germany; 9 Missouri Botanical Garden, St. Louis, 63110 Missouri USA

## Abstract

This comment raises concerns regarding the way in which a new European directive, aimed at reaching higher renewable energy targets, treats wood harvested directly for bioenergy use as a carbon-free fuel. The result could consume quantities of wood equal to all Europe’s wood harvests, greatly increase carbon in the air for decades, and set a dangerous global example.

In January of this year, even as the Parliament of the European Union admirably voted to double Europe’s 2015 renewable energy levels by 2030, it also voted to allow countries, power plants and factories to claim that cutting down trees just to burn them for energy fully qualifies as low-carbon, renewable energy. It did so against the written advice of almost 800 scientists that this policy would accelerate climate change^1^. This Renewable Energy Directive (RED) is now finalized. Because meeting a small quantity of Europe’s energy use requires a large quantity of wood, and because of the example it sets for the world, the RED profoundly threatens the world’s forests.

Makers of wood products have for decades generated electricity and heat from wood process wastes, which still supply the bulk of Europe’s forest-based bioenergy^2,3^. Although burning these wastes emits carbon dioxide, it benefits the climate because the wastes would quickly decompose and release their carbon anyway. Yet nearly all such wastes have long been used^4^.

Over the last decade, however, due to similar flaws in the 2008 RED, Europe has expanded its use of wood harvested to burn directly for energy, much from U.S. and Canadian forests in the form of wood pellets. Contrary to repeated claims, almost 90% of these wood pellets come from the main stems of trees, mostly of pulpwood quality, or from sawdust otherwise used for wood products^5^.

## Greenhouse gas effects of burning wood

Unlike wood wastes, harvesting additional wood just for burning is likely to increase carbon in the atmosphere for decades to centuries^6–16^. This effect results from the fact that wood is a carbon-based fuel whose harvest and use are inefficient from a greenhouse gas (GHG) perspective. Typically, around one third or more of each harvested tree is contained in roots and small branches that are properly left in the forest to protect soils but that decompose and release carbon. Wood that reaches a power plant can displace fossil emissions but per kWh of electricity typically emits 1.5x the CO_2_ of coal and 3x the CO_2_ of natural gas because of wood’s carbon bonds, water content (Table 2.2 of ref. 17) and lower burning temperature (and pelletizing wood provides no net advantages) (Supplementary Note 1)^6,16^.

Allowing trees to regrow can reabsorb the carbon, but for some years a regrowing forest typically absorbs less carbon than if the forest were left unharvested, increasing the carbon debt. Eventually, the regrowing forest grows faster and the additional carbon it then absorbs plus the reduction in fossil fuels can together pay back the carbon debt on the first stand harvested. But even then, carbon debt remains on the additional stands harvested in succeeding years, and it takes more years for more stands to regrow before there is just carbon parity between use of wood and fossil fuels. It then takes many more years of forest regrowth to achieve substantial GHG reductions.

The renewability of trees, unlike fossil fuels, helps explain why biomass can eventually reduce GHGs but only over long periods. The amount of increase in GHGs by 2050 depends on which and how forests are ultimately harvested, how the energy is used and whether wood replaces coal, oil or natural gas. Yet overall, replacing fossil fuels with wood will likely result in 2-3x more carbon in the atmosphere in 2050 per gigajoule of final energy (Supplementary Note 2). Because the likely renewable alternative would be truly low carbon solar or wind, the plausible, net effect of the biomass provisions could be to turn a ~5% decrease in energy emissions by 2050 into increases of ~5–10% or even more (Supplementary Note 2).

## Consequences for forests

The implications for forests and carbon are large because even though Europe harvests almost as much wood as the US and Canada combined, these harvests could only supply ~5.5% of its primary energy and ~4% of its final energy. If wood were to supply 40% of the additional renewable energy—an uncertain but plausible level—the wood volumes required would equal all of Europe’s wood harvest (Supplementary Note 3). In fact, the RED sets a goal to increase by 10% renewable energy for heat, sourced overwhelmingly from wood, which would likely by itself use ~50% of Europe’s present annual wood harvest^18,19^. European Commission planning documents projected somewhat smaller roles for bioenergy based on lower renewable energy targets, but they scale up to ~55–85% of Europe’s wood harvest at the larger target ultimately adopted (Supplementary Note 4). Supplying this level of wood will probably require expanding harvests in forests all over the world.

The global signal may have even greater effects on climate and biodiversity. At the last global climate conference (UNFCCC-COP 23, Bonn 2017), tropical forest countries and others, including Indonesia and Brazil, jointly declared goals “to increase the use of wood … to generate energy as part of efforts to limit climate change”^20,21^. Once countries and powerful private companies become invested in such efforts, further expansion will become harder to stop. The effect can already be seen in the United States, where Congress in both 2017 and 2018 added provisions to annual spending bills declaring nearly all forest biomass carbon free—although environmentalists have so far fought to limit the legal effects to a single year^22,23^. If the world met just an additional 2% of global primary energy with wood, it would need to double its industrial wood harvests (Table 1).

**Table 1:** Wood harvest energy and potential demands

Region	Roundwood production	Harvest volume 2015 (10^6^ m^3^)	Energy content of harvested wood (EJ)	Total primary energy consumption 2015 (EJ)^a^	Potential % of present primary energy supplied by 2015 roundwood harvests	Plausible primary wood biomass energy required by new directive (EJ)^b^	% of 2015 wood harvest plausibly required for expanded bioenergy in 2030^c^
Europe	Industrial	333	3	70	4.3%	3.9	130%
	Total	428	3.85	70	5.6%	3.9	101%
World	Industrial	1826	17.9	571	2.1%		
	Total	3688	36.1	571	4.2%		

^a^Based on estimate of 0.49 tDM/m^3^ for the World and 0.45 tDM/m^3^ for Europe and 20 GJ/tDM (Supplementary Methods)

^b^Assumes roundwood supplies 40% of mandated increase in Europe’s final renewable energy from 2015–2030, which would be mandated by RED, 35% used for bioelectricity at 25% efficiency and 65% for heat at 85% efficiency (Supplementary Note 3)

^c^Also assumes Europe meets 32% target increase in European economy-wide energy efficiency from 2007 levels by 2030 (Supplementary Note 3)

## Why the RED sustainability criteria are insufficient

Unfortunately, various sustainability conditions in the RED would have little consequence. For example, one repeated instruction is that harvesting trees should occur sustainably, but sustainable does not equal low carbon (Supplementary Note 5). Perhaps the strictest version of sustainability, often defended as a landscape approach, claims GHG reductions so long as harvest of trees in a country (or just one forest) does not exceed the forest’s incremental growth^24–27^. Yet, by definition, this incremental growth would otherwise add biomass, and therefore carbon storage to the forest, holding down climate change^28^. This carbon sink, in large part due to climate change itself, is already factored into climate projections and is not disposable. Harvesting and burning this biomass reduces the sink and adds carbon to the air just like burning any other carbon fuel. The directive only requires forests to maintain existing carbon stocks in limited circumstances, but given the size of the global forest sink, even applying such a rule everywhere would still allow global industrial wood harvests to more than triple (Supplementary Note 6)^29,30^.

The directive also repeatedly cites a goal to preserve biodiversity, but its provisions will afford little protection. Prohibitions on harvesting wood directly for bioenergy apply only to primary forests—a small share of global forests (Supplementary Note 5). In addition, any forests could be cut to replace the vast quantities of wood diverted from existing managed forests to bioenergy.

Some argue that increasing carbon in the atmosphere for decades is fine so long as reductions eventually occur, but timely mitigation matters. More carbon in the atmosphere for decades means more damages for decades, and more permanent damages due to more rapid melting of permafrost, glaciers and ice-sheets, and more packing of heat and acidity into the world’s oceans. Recognizing this need, the EU otherwise requires that GHG reductions occur over 20-years, but that timing does not apply to forest biomass (Supplementary Note 5).

Instead, the directive incorporates the view that forest biomass is inherently carbon neutral if harvested sustainably (Supplementary Note 5). Although the RED requires that bioenergy generate large greenhouse gas reductions, its accounting rules ignore the carbon emitted by burning biomass itself (Annex VI, section C, par 13 in ref. 31). They only count GHGs from trace gases and use of fossil fuels to produce the bioenergy, which is like counting the GHGs from coal-mining machinery but not from burning the coal.

The main new Commission thinking, reflected in the sustainability provisions, is that bioenergy rules do not need to count plant carbon so long as countries that supply the wood have commitments related to land use emissions under European rules or the Paris accord (RED, Article 26, point (6)(1)(ii)) (Supplementary Note 5). But this thinking repeats the confusion that occurred at the time of the Kyoto Protocol between rules designed only to count global emissions and laws designed to shape national or private incentives^32^. Under accounting rules for the UN Framework Convention on Climate Change (UNFCCC), countries that burn biomass can ignore the resulting energy emissions because the countries that cut down the trees used for the biomass must count the carbon lost from the forest. Switching from coal to biomass allows a country to ignore real energy emissions that physically occur there, but the country supplying the wood must report higher land use emissions (at least compared to the no-bioenergy alternative). The combination does not make bioenergy carbon free because it balances out global accounting, the limited goal of national reporting.

But this accounting system does not work for national energy laws. If a country’s laws give its power plants strong financial incentives to switch from coal to wood on the theory that wood is carbon-neutral, those power plants have incentives to burn wood regardless of the real carbon consequences. Even if a country supplying the wood reports higher land use emissions through the UNFCCC, that carbon is not the power plant’s problem. Only if all potential wood-supplying countries imposed a carbon fee on the harvest of wood, and this fee equaled Europe’s financial incentive to burn it, would European power plants have a financial reason to properly factor the carbon into their decisions. No country has done that or seems likely to do so.

In fact, few countries have any obligation to compensate for reduced carbon in their forests because few countries have adopted quantitative goals in the land use sector as part of the Paris accord^33^. Even if countries did try to make up for reduced forest carbon due to bioenergy with additional mitigation of some kind, all Europe would achieve is a requirement that its consumers pay more to do something harmful for the climate so that other countries could then spend additional money to compensate.

Europe has also created a kind of reverse REDD + strategy by treating forest and all other biomass as carbon neutral in its Emissions Trading System, which limits emissions from power plants and factories. While the not yet realized hope behind REDD + is to reward countries for preserving carbon in forests, this bioenergy policy means forest owners can be rewarded for the carbon in their trees—so long as they cut them down and sell them for energy. The higher the price of carbon rises, the more valuable cutting down trees will become. Strangely, this policy also undermines years of efforts to save trees by recycling used paper instead of burning it for energy. Even as recycling polices push consumers to save trees, this policy will encourage others to burn them.

## Alternative low carbon energy sources

Alternatives include various forms of solar power, which typically generate at least 100 times more useable energy per hectare than bioenergy even on good land—and even more on dry lands and rooftops^34,35^. Possible future limits on solar if storage does not evolve cannot justify bioenergy today. With solar costs already dropping below $US 0.02/kWh in some world locations, and offshore wind in Europe below $US0.06, solar and wind have many economic advantages over bioenergy, particularly for electricity, even with bioenergy’s incorrect GHG accounting^36^. Unfortunately, these advantages are unlikely to fully negate the political and occasional economic benefits enabled by flawed climate accounting of simply replacing fossil fuels with wood.

Although some scientists support this use of forests^24,26,27^, and the IPCC has found it difficult to speak clearly about biomass in the face of different views (see Appendix 11.13 in ref. 37), the fact that ~800 scientists came forward provides hope of a clearer and stronger message from the scientific community. The fate of the biosphere appears at stake. Individual European countries still have discretion to pursue alternatives to forest biomass. Whatever their fields, all scientists who care should educate themselves, overcome a natural reluctance to venture into a separate and controversial field, speak with great clarity and hold public institutions to account.

## Electronic supplementary material

Supplementary Information

## Data Availability

All data used or calculated for this comment are presented in tables in the main text or supplement or are available from publicly available sources cited.
