# Supplementary material for: Europe’s renewable energy directive poised to harm global forests
Source: Nat Commun. 2018 Sep 12;9:3741. doi: 10.1038/s41467-018-06175-4 (PMC6135810; doi:10.1038/s41467-018-06175-4)
Supplement: Supplementary file 1 — Supplementary Information [file 41467_2018_6175_MOESM1_ESM.pdf]

SUPPLEMENTARY INFORMATION

Europe's Renewable Energy Directive Poised to Harm Global Forests

Searchinger et al.

Supplementary Methods

The sources used to prepare table 1 are as follows. Total roundwood production of the World from FAOSTAT: http://www.fao.org/faostat/en/#data/FO; total roundwood production in Europe (EU-28) from Eurostat: http://ec.europa.eu/eurostat/statistics-explained/index.php/Wood_products_-_production_and_trade. (Note, the European roundwood production number from FAOSTAT is larger at 459 million cubic meters. We do not know the explanation for the difference. That would increase total wood harvest to 4.1 EJ.); Conversion from cubic meters to dry matter assumes 1/3 coniferous and 2/3 non-coniferous based on FAOSTAT, and .411 tonnes DM/cubic meter coniferous and .523 tonnes DM/cubic meter non-coniferous wood based on Table 5 in Miles and Smith[1]; Calculation of energy content of wood assuming an energy content of 20 GJ/t of dry matter; Global total primary energy consumption is taken from International Energy Agency[2]. Total primary energy consumption in Europe (EU-28) is taken from the European Commission[3].

Supplementary Table 1: Calculation of the share of primary energy consumption that could be produced from current total or industrial roundwood production.

	Year	**Industrial** roundwood production (million cubic meters)	Avg. weight in dry matter per cubic meter	Total wood in dry matter volume (millions tonnes)	Energy content of wood total (EJ)	Total Primary Energy Consumption (EJ)	% of primary energy consumption
World	2015	1,826	0.49	895	17.9	571	2.1%
Europe	2015	333	0.45	150	3.0	70	4.3%
		**Total** roundwood production					
World	2015	3,688	0.49	1807	36.1	571	4.2%
Europe	2015	428	0.45	193	3.9	70	5.5%

Supplementary Note 1 – Smokestack Emissions Wood v. Fossil Fuels

The additional carbon released burning wood per kWh compared to coal or natural gas is one part of the explanation of why using wood will increase greenhouse gas emissions for decades. The figure is a function of (a) the higher carbon content of wood than fossil fuels per kWh of potential gross energy, and (b) the lower conversion efficiencies. The figure that wood generates smokestack emissions 50% more than burning coal and more than three times that of natural gas are based on the numbers used in Laganière et al.[4] except we use IPCC emission factors for the net calorific value of fuel, which are more favorable to wood than those used in Laganière. Some papers claim higher conversion efficiencies for burning wood, up to roughly 30%, but coal and natural gas conversion efficiencies are also often much higher[5].

Wood pellets can burn at higher temperature and produce electricity with greater efficiency, but their production creates more greenhouse gas emissions and involves large

process losses along the way. For example, Röder et al.[6] calculate that 38% of the wood is lost at various stages of handling, processing or use, including 18% lost to supply heat for pellet drying if fossil fuels are not used for that process. (If fossil fuels are used, they cause their own emissions.) These losses in wood for fuel or other added emissions will roughly cancel out the benefits of converting wood to wood pellets depending on the precise range of emissions and ultimate burning efficiencies. For example, Sterman et al.[7] , supplementary information, p. 27, calculate that burning wood pellets for electricity generates 183% of the emissions of burning coal when considering the whole supply chain, ignoring losses of terrestrial carbon for this part of the calculation, although this paper uses a relatively low conversion efficiency for electricity production.

Supplementary Table 2: Typical Smokestack Emissions for Electricity Production.

	Kg CO_2eq released/ GJ net calorific value of fuel*	Kg CO_2eq/ kWh net calorific value of fuel	Conversion efficiency to electricity**	Total emissions Kg CO_2eq per kWh	Ratio of wood to alternative fossil fuel
Wood	112.00	0.40	0.26	1.55	-
Coal	94.60	0.34	0.33	1.03	150%
Oil	71.10	0.26	0.35	0.73	212%
Natural gas	64.20	0.23	0.45	0.51	302%

Sources:
* IPCC, 2006 IPCC Guidelines for National Greenhouse Gas Inventories, Volume 2 Stationary Installations, Table 2.2 (ref. 8)
** Laganière et al.[4]
Note that often wood is co-fired with coal. In that case, the total burning efficiency of the mix can be closer to that of coal, e.g., 30-31%, but the net contribution to final kWh of wood is even lower because it lowers the conversion efficiency of the coal.

Supplementary Note 2 – GHG Increases by 2050 from Wood Bioenergy

Many papers have calculated the greenhouse gas consequences of harvesting additional trees for bioenergy as cited in the main text. The precise results vary greatly with the type of forest harvested, the harvest regime, the fuel use (biomass conversion efficiencies come closer to those of fossil fuels when used for heat than for electricity), and the type of fossil fuel displaced. Most frequently, papers report payback or parity periods, which are the periods trees must be allowed to regrow before the net effect on greenhouse gases (incorporating both changes in terrestrial carbon and reductions in fossil carbon emissions) are equivalent for use of biomass and the fossil fuel alternative. These payback periods are sometimes reported assuming a single stand harvest in one year, which, for reasons discussed in the main text, are shorter than for a sustained harvest. In general, if the payback period is 60 years, then the emissions after 30 years would typically be at least and probably more than double those of fossil fuels (because the payoff of debt should be faster in the second thirty-year period than the first).

Using Laganière et al.[4], the parity point for harvest and use of otherwise standing Canadian trees (as opposed to salvaged trees or residues) and allowing the same forests to regrow is always longer than 90 years for two classes of forests and has a shortest period of roughly 70 years for the shortest rotation forests even when replacing coal and using wood

for heat. All harvests even for heat have payback periods of more than 100 years when replacing natural gas. Sixty-year payback periods would imply at least a doubling of emissions and 90 years would imply at least a tripling of emissions by 2050. Mitchell et al.[9] also examine a wide variety of forest types and uses. Although a few possible scenarios have payback periods as fast as 30 years (which means emissions would only equal fossil fuel use after that period), the overwhelming majority are well more than 60 years, and most are well more than 90 years. Holtsmark[10] found slightly more than a doubling of atmospheric carbon for harvesting from boreal forests in Europe to replace coal at around 40 years. Based on these figures, a doubling or tripling of emissions is possible, or even more, depending on the ultimate mix of forest sources and uses.

One possible reason for even higher concern is that plantation forests provide less than half and probably only around one third of wood harvests today[11]. The fastest payback times occur assuming that wood harvests come from fast-growing plantations that would otherwise not be harvested. This assumption of non-harvest seems highly improbable for these highly managed forests. Yet if this wood is diverted to bioenergy use instead of wood products, replacements need to come from slower growing, natural forests with much longer pay-back periods.

Final energy consumption in the EU-28 in 2015 was 1,084 MTOE or 45.4 EJ. With a share of 16.7%, renewables contributed 7.6 EJ of final energy consumption[12]. The three parts of the European Union (the Commission, Parliament and Council of States) have now agreed on a renewable energy target of 32% for 2030. That requires that the share of renewables in final energy would have to grow from 2015 to 2030 by 15.3% compared to 2015. In Supplementary Note 3, we explain our reasoning that additional forest biomass could plausibly supply 40% of this increase or an additional 6.1% of Europe's final energy in 2030 or ~2.5 EJ. After deducting the portion of Europe's existing final energy use that is attributable to renewables, we obtain 37.8 EJ, so this additional final energy from forest biomass would replace 6.6% of existing final energy from non-renewable sources.

Assuming broadly (a) that this 40% would otherwise be replaced by essentially carbon neutral solar or wind, and (b) that the greenhouse gas emissions from this energy displaced would otherwise have emissions at the European average energy emissions rate, the RED would otherwise save ~6.6% of Europe's existing energy emissions. Assuming instead sources of biomass that double or triple emissions by 2050, the RED provisions result in a ~6.6 to ~13.2 percent increase. Because these numbers make many broad assumptions, we present them as 5% and 10% changes to avoid conveying a false sense of precision, and to also build in an additional level of conservatism. The actual effect will depend on many factors including the actual amount of forest biomass used, what energy sources it replaces, and both the precise direct and indirect sources of wood (including wood harvested to replace wood diverted to bioenergy use that would otherwise supply wood products). We believe this estimate provides one rough, plausible estimate of the emissions consequences of the forest biomass provisions of the RED.

Supplementary Note 3 – Share of European Wood Harvest Needed

Using data from Tables 2.1, 2.2 and 2.3 from ref. 13, total growth of final renewable energy in the EU28 from 2005 to 2015 was 78,248 ktoe, of which 46,430 ktoe was from

some source of biomass, or 59%. Only around 52% of this biomass was wood, so increased use of wood provided around 30% of the growth in renewable energy in this period. However, caps on crop-based liquid biofuels in the directive at existing levels or with small gains are likely to reduce the contribution in coming years from those sources, which leaves more future bioenergy gains to be achieved by wood. The RED also establishes a separate goal of a 1% increase each year in renewable energy in heat, of which the vast majority comes currently from biomass[14]. One analysis found that meeting this target would likely require an additional 51 Mtoe, or 2.13 EJ (ref. 14, Table 4-1), which itself is roughly one third of the increased renewable energy, and based on that study's estimate of required 236 million cubic meters of roundwood equivalent, would itself require 55% of existing European roundwood harvest. Putting these various trends and requirements together, we find it plausible that forest biomass would supply 40% of Europe's renewable energy. It could be less but it could also be much more.

Supplementary note 2 explains how the RED requires an increase in final renewable energy by 15.3% compared to 2015, but the amount of primary biomass energy that requires depends in part on how the biomass is used. We here assume 35% of biomass is used for electricity at 25% efficiency and 65% is used for heat at 85% efficiency, which generates a total conversion efficiency from primary to final energy of 64% (efficiencies of different bioenergy conversion technologies were taken from Edwards et al.[15]). The quantity also depends on how much final energy Europe actually consumes in 2030. After debating different targets, Europe has agreed on a target of 32.5% increase in economy-wide energy relative to 2007 although the target is not binding[16], which we calculate would lead to total EU final energy consumption of 43.25 EJ based on extrapolations at this efficiency from EU projections of economic growth in ref. 3. An increase of 15.3% in final renewable energy would require an increase of 6.24 EJ of final renewable energy at the 32.5% agreed upon efficiency target. If 40% is supplied by wood at 64% efficiency that would require 3.9 EJ of wood, equal to 101% of Europe's total existing wood harvest of 3.85 EJ of roundwood (Supplementary Methods).

Supplementary Note 4 – Harvest Increases Implied by European Commission Estimates

The European Commission made its own estimates of future renewable biomass to meet its originally proposed targets of the RED. One estimate using the Commission's PRIMEs modeling shows an increase of 43 Mtoe from 2013 to 2030, or 1.8 EJ, which would be 46% of current roundwood harvest in Europe[17,18]. Green-X modeling projects an increase in the share of energy from biomass in primary energy consumption from roughly 8% in 2013 to roughly 12% in 2030. If that increase comes from wood, it implies an increase of 2.2EJ or 56% of existing roundwood harvest assuming the European economy-wide 32.5% energy efficiency increase in 2030 compared to 2007 as discussed above. However, these model runs assumed a 27% renewable energy target in 2030 and different energy efficiency increases for the whole EU economy. Scaling proportionately for a 32% renewable energy target and a 32.5% "increase" in economy-wide energy efficiency relative to 2007 would require 15.3 EJ more than the 2015 renewable energy use of 16.7%. That is a 48.5% larger increase in renewable energy than implied by the increases in renewable energy assumed by the EU modeling. Although actually running the models with the 32% renewable energy

target might generate somewhat different results, scaling up the increase in biomass proportionately to the larger increase in renewables, the biomass increase rises to 2.2 EJ for PRIMES modeling and 3.3 EJ for Green-X modeling, If these increases come from wood, the additional wood would equal 56-85% of existing European wood harvest.

Supplementary Note 5 – RED Sustainability Criteria

The Renewable Energy Directive contains much language setting forth restrictions on biomass that qualifies for the directive and that are referred to as sustainability standards, and we discuss the most important ones in the text. None of these provisions would significantly reduce impacts on forests overall, and we outline them here. There were small differences in the language originally proposed by the European Commission and that enacted by the European Parliament. In the cases of original disagreement, the language quoted here cites language agreed upon by negotiators for the Commission, the Parliament and the European Council, which was released on June 21, 2018[19].

Article 26, paragraph 7, provides requirements that the use of biomass reduce greenhouse gas emissions by at least 50% for installations in operation before 2015, and higher for installations coming on line later. However, the methodology for calculating greenhouse gas emission are set forth in Appendix V for liquid biofuels and Appendix VII for factories and power plants (among other stationary installations). They explicitly exclude the carbon released by burning the biomass (Appendix V, Part C. par. 13; Appendix VI, Part C., par. 13): “Emissions of CO_2 from fuel in use, e_u, shall be taken to be zero for biomass fuels.” As a result, the emissions that count are only the fossil emissions and trace gases generated in harvesting and production process, not the carbon in the biomass itself, which is what it means for a fuel to be carbon neutral. This can also be seen in tables providing default values for the use of biomass fuels (Annex VI C).

The appendices (V & VI for the different types of bioenergy) also provide for counting emissions from carbon stock changes resulting from land use change. These emissions are to be counted if conversion of land to crop production, including bioenergy crops, involves converting forests and other high carbon lands. Emissions from land use change must be amortized over 20 years, meaning that each mega joule of energy in fuel is assigned greenhouse gases equal to the emissions from land use change of a hectare divided by twenty and divided by the mega joules that are produced from biomass grown on that hectare each year. But land use changes only apply to the direct conversion from forest to cropland or grassland, not merely from the harvest of wood on existing forest (Annex VIII Part B).

There are provisions restricting the use of biomass from some high carbon stocks, but they apply only to conversions from other land uses for the production of “agricultural raw material” (Article 26, par. 70 of the Proposed RED), and therefore do not apply to the harvest of forests.

The directive includes numerous general references to the importance of sustainability. For example, paragraph 76 of Article 26 provides that “woody raw material should come only from forests that are harvested in accordance with the principles of sustainable forest management developed under international forest processes such as Forest Europe and are

implemented through national laws or the best management practices at the forest sourcing level." The text also states: "Operators should take the appropriate steps in order to minimize the risk of using unsustainable forest biomass for the production of bioenergy." These kinds of provisions do not restrict the quantity of forest harvest, nor do they even appear to have much implication for major harvest methods – for example, clear-cutting will typically comply with best management practices requirements.

There are also some more specific provisions whose principal significance is that forests be allowed and able to regenerate (Article 26, paragraph 5). The two most specific of these are (a)(i), which requires "forest regeneration of harvested", and (a)(v), which requires that "harvesting maintains or improved the long-term production capacity of the forest". This language is designed to assure renewability. The second type of provisions quoted is ambiguous. It is not clear if it restricts the volume of harvest at all as a forest can be harvested without eliminating its long-term production capacity. It is unlikely to be interpreted as holding harvesting to the incremental growth, but if it were interpreted in that way, then it still does not substantially restrict global harvest because it allows elimination of the national, regional or global forest carbon sink as discussed in the main text.

Article 26, paragraph 6 sets forth certain provisions for the source of forest biomass related to land use management. The basic requirement is that forest biomass comes from a country that "has submitted a Nationally Determined Contribution (NDC) to the United Nations Framework Convention on Climate Change (UNFCCC), covering emissions and removals from agriculture, forestry and land use which ensures that either changes in carbon stock associated with biomass harvest are accounted towards the country's commitment to reduce or limit greenhouse gas emissions as specified in the NDC." As discussed in the main text, this provision will not discourage use of wood that increases greenhouse gas emissions both because most country commitments are quite weak and because all the provision might require at most is a requirement that countries compensate for lost carbon in the forest by additional mitigation elsewhere. If a country does not participate in the Paris accord, e.g., the United States at this time, biomass can still qualify. According to the agreed upon text, "When evidence referred to in the first subparagraph is not available, the biofuels, bioliquids and biomass fuels produced from forest biomass shall be taken into account for the purposes referred to in points (a), (b) and (c) of paragraph 1 if management systems are in place at the forest sourcing level to ensure that carbon stocks and sinks levels in the forest are maintained or strengthened over the long term." Although this language may require maintenance of carbon sinks in a forest, not merely carbon stocks, a supplier will be able to meet it just by showing that the forest material harvested would otherwise have been harvested because that cannot reduce a sink. This language might therefore restrict or limit new harvesting in an area but will allow diversion of wood from other uses. Doing so would therefore not restrict the global significance of the additional wood demand.

The sustainability criteria include many references to biodiversity. The most specific of these is Commission language, which provides that "areas of high conservation value, including wetlands and peatlands, are protected," and which the Parliamentary version altered to apply to areas "legally designated for nature conservation purposes". In addition, Recital 71 provides that biomass should not originate from areas classified by the UN Food

and Agriculture Organization as "primary forests," or are protected by national laws. However, FAO defines primary forests as follows: "Naturally regenerated forest of native species, where there are no clearly visible indications of human activities and the ecological processes are not significantly disturbed."[20]. This is a highly restrictive category of forests.

In addition, the language regarding biodiversity or even quantities of forest harvest applies only to the forest area being harvested. One of the major effects of the directive is likely to be the diversion of wood from already highly managed forests and many other disturbed forests, which increases the global demand for wood. Nothing in the directive restricts what happens to even the most biologically diverse forest as a result of this displacement of wood from more managed forests.

Supplementary Note 6 – Biomass and Global Forest Carbon Sink

Pan et al.[21] estimates a net, global terrestrial carbon sink of 1.04 GtC. Assuming 50% carbon, that translates into ~2.1 GtDM, which is equal to ~230% of existing industrial wood harvest (~0.9 Gt) as shown in Supplemental Table 1. As a result, existing industrial wood harvest (harvests not used for traditional firewood and charcoal) could be more than tripled (from ~0.9 Gt to ~3Gt) without eliminating the net global forest sink and would therefore be allowable under the sustainability principle of harvesting only the annual incremental growth of forest.

Typical approaches to sustainability suggest netting at a country not global level, which would greatly expand this potential harvest with a rule of harvesting only incremental growth and just up to the elimination of the carbon sink. Using country-netting, countries with larger sinks could increase their harvests for bioenergy and still meet this sustainability standard without having to maintain a sink to compensate for the net forest carbon losses in other countries. As a result, the total sink just of countries with sinks exceeds the ~2.1GtDM global, net sink from all countries. Our estimate of at least a three-fold increase in allowable, industrial harvest is therefore conservative.

Supplementary References

1. Miles, P. D. & Smith, W. B. *Specific gravity and other properties of wood and bark for 156 tree species found in North America*. (U.S. Department of Agriculture, Forest Service, Northern Research Station, 2009).
2. International Energy Agency. World Energy Balances 2017: Overview. (2017).
3. European Commission. *IMPACT ASSESSMENT Accompanying the document Proposal for a Directive of the European Parliament and of the Council amending Directive 2012/27/EU on Energy Efficiency*. (2016).

4. Laganière, J., Paré, D., Thiffault, E. & Bernier, P. Y. Range and uncertainties in estimating delays in greenhouse gas mitigation potential of forest bioenergy sourced from Canadian forests. *GCB Bioenergy* **9,** 358–369 (2017).
5. Brack, D. *Woody Biomass for Power and Heat Impacts on the Global Climate*. (Chatham House, 2017).
6. Röder, M., Whittaker, C. & Thornley, P. How certain are greenhouse gas reductions from bioenergy? Life cycle assessment and uncertainty analysis of wood pellet-to-electricity supply chains from forest residues. *Biomass and Bioenergy* **79,** 50–63 (2015).
7. Sterman, J. D., Siegel, L. & Rooney-Varga, J. N. Does replacing coal with wood lower CO2 emissions? Dynamic lifecycle analysis of wood bioenergy. *Environ. Res. Lett.* **13,** 015007 (2018).
8. IPCC. *IPCC Guidelines for National Greenhouse Gas Inventories*. (Published: IGES, 2006).
9. Mitchell, S. R., Harmon, M. E. & O'Connell, K. E. B. Carbon debt and carbon sequestration parity in forest bioenergy production. *Glob. Change Biol. Bioenergy* **4,** 818–827 (2012).
10. Holtsmark, B. Harvesting in boreal forests and the biofuel carbon debt. *Climatic Change* **112,** 415–428 (2012).
11. Jürgensen, C., Kollert, W. & Lebedys, A. *Assessment of industrial roundwood production from planted forests. FAO Planted Forests and Trees Working Paper FP/48/E.* (2014).
12. eurostat. *Share of renewable energy in gross final energy consumption*. http://ec.europa.eu/eurostat/web/products-datasets/-/t2020_31 (2018).
13. European Environment Agency. *Renewable energy in Europe – 2017 Update. Recent growth and knock-on effects.* (2017).

14. Hennenberg, K., Böttcher, H., Fehrenbach, H. & Bischoff, M. *Short analysis of the RED 2009, the iLUC Directive 2015 and the 2016 RED proposal regarding implications for nature protection*. (Oeko-Institute and IFEU, 2017).
15. Edwards, R., Larivé, J. F., Rickeard, D. & Weindorf, W. *Well-to-Tank Report Version 4.a: JEC Well-to-Wheels Analysis*. (European Commission, Joint Research Centre, Institute for Energy and Transport, 2014).
16. European Commission. Energy efficiency first: Commission welcomes agreement on energy efficiency. (2018). Available at: http://europa.eu/rapid/press-release_STATEMENT-18-3997_en.htm. (Accessed: 14th August 2018)
17. European Commission. *IMPACT ASSESSMENT Sustainability of Bioenergy*. (2016).
18. PricewaterhouseCoopers EU Services EESV's consortium. *Sustainable and optimal use of biomass for energy in the EU beyond 2020*. (2017).
19. Council of the European Union. *Proposal for a Directive of the European Parliament and of the Council on the promotion of the use of energy from renewable sources - Analysis of the final compromise text with a view to agreement*. Available at: http://data.consilium.europa.eu/doc/document/ST-10308-2018-INIT/en/pdf (2018).
20. UN Food and Agriculture Association. *Terms and Conditions FRA 2020*. (UN Food and Agriculture Association, 2018).
21. Pan, Y. *et al.* A Large and Persistent Carbon Sink in the World's Forests. *Science* **333,** 988–993 (2011).
